# In vivo efficacy of the histone deacetylase inhibitor suberoylanilide hydroxamic acid in combination with radiotherapy in a malignant rhabdoid tumor mouse model

**DOI:** 10.1186/1748-717X-7-52

**Published:** 2012-03-29

**Authors:** Markus Thiemann, Susanne Oertel, Volker Ehemann, Wilko Weichert, Albrecht Stenzinger, Marc Bischof, Klaus-J Weber, Ramon Lopez Perez, Uwe Haberkorn, Andreas E Kulozik, Jürgen Debus, Peter E Huber, Claudia Battmann

**Affiliations:** 1 Department of Radiation Oncology, University of Heidelberg, INF 600, 69120 Heidelberg, Germany; 2 Institute of Pathology, University of Heidelberg, 69120 Heidelberg, Germany; 3 Department of Radiation Oncology, German Cancer Research Center, 69120 Heidelberg, Germany; 4 Department of Nuclear Medicine, University of Heidelberg and DKFZ, 69120 Heidelberg, Germany; 5 Department of Pediatric Oncology, Hematology and Immunology, University Children's Hospital of Heidelberg, 69120 Heidelberg, Germany

**Keywords:** Malignant rhabdoid tumor, In vivo, Radiotherapy, Histone deacetylase inhibition, Suberoylanilide hydroxamic acid

## Abstract

**Purpose:**

Histone deacetylase inhibitors are promising new substances in cancer therapy and have also been shown to sensitize different tumor cells to irradiation (XRT). We explored the effect as well as the radiosensitizing properties of suberoylanilide hydroxamic acid (SAHA) in vivo in a malignant rhabdoid tumor (MRT) mouse model.

**Methods and material:**

Potential radiosensitization by SAHA was assessed in MRT xenografts by analysis of tumor growth delay, necrosis (HE), apoptosis (TUNEL), proliferation (ki-67) and γH2AX expression as well as dynamic ^18^F-Fluorodeoxyglucose Positron Emission Tomography (^18^F-FDG -PET) after treatment with either SAHA alone, single-dose (10 Gy) or fractionated XRT (3 × 3Gy) solely as well as in combination with SAHA compared to controls.

**Results:**

SAHA only had no significant effect on tumor growth. Combination of SAHA for 8 days with single-dose XRT resulted in a higher number of complete remissions, but failed to prove a significant growth delay compared to XRT only. In contrast fractionated XRT plus SAHA for 3 weeks did induce significant tumor growth delay in MRT-xenografts.

The histological examination showed a significant effect of XRT in tumor necrosis, expression of Ki-67, γH2AX and apoptosis. SAHA only had no significant effect in the histological examination. Comparison of xenografts treated with XRT and XRT plus SAHA revealed a significantly increased γH2AX expression and apoptosis induction in the mice tumors after combination treatment with single-dose as well as fractionated XRT. The combination of SAHA with XRT showed a tendency to increased necrosis and decrease of proliferation compared to XRT only, which, however, was not significant. The ^18^F-FDG-PET results showed no significant differences in the standard uptake value or glucose transport kinetics after either treatment.

**Conclusion:**

SAHA did not have a significant effect alone, but proved to enhance the effect of XRT in our MRT in vivo model.

## Introduction

The aim of the present study was to evaluate the in vivo efficacy of the HDACi SAHA in combination with XRT in MRT xenotransplants.

MRT and their central nervous system counterpart atypical teratoid/rhabdoid tumors are rare and highly malignant neoplasms that primarily occur in young children. Emerging evidence suggests an important role for radiotherapy to achieve long-term survival. Nevertheless even after toxic multimodal treatment including radiotherapy outcome is generally poor and new therapy options are urgently needed [1,2]. Therefore interest in adjuvant agents that selectively augment the response of MRT to radiation and thus increase the therapeutic ratio is high. Histone deacetylase inhibitiors (HDACi) are under investigation in anti-cancer treatment. They work by epigenetic regulation of gene expression, induce cell growth arrest, apoptosis as well as terminal differentiation. Clinical trials so far indicate moderate toxicity and favorable safety profiles. Even though in single-agent treatments only moderate improvements in outcome were observed [3], the combination of HDACi with other therapy modalities like radiotherapy has been reported to improve the therapeutic effect in many tumor entities in cell lines and xenograft tumor models [4-7]. We recently reported that the HDACi suberoylanilide hydroxamic acid (SAHA) radiosensitized pediatric sarcoma and rhabdoid tumor cell-lines in vitro through increase of apoptosis and regulation of the cell cycle as well as down-regulation of DNA-repair proteins [8]. Knipstein et al. reported that histone deacetylase inhibitors decrease proliferation and potentiate the effect of ionizing radiation in two further rhabdoid tumor cell lines [9].

Our data as well as the data of other groups showed that the radiosensitization through HDACi is associated with increased expression of γH2AX in treated tumor cell lines, supporting the theory that changes in XRT-induced DNA damage repair plays a role in the radiosensitization of tumor cells by HDACi [10]. γ-H2Ax expression was therefore investigated in the treatment xenograft specimen along with proliferation, apoptosis and necrosis as further possible underlying mechanisms.

Measurement of glucose metabolism with positron emission tomography (PET) has become an important imaging modality in the non-invasive evaluation and monitoring of malignant diseases. In sarcoma patients, ^18^F-Fluorodeoxyglucose Positron Emission Tomography (^18^F-FDG PET) has been shown to be useful in staging, therapy monitoring, and detection of relapse [11]. The use of PET/CT using F-FDG in rare tumors like MRT is unclear. Therefore, we were interested in the correlation of glucose metabolism with clinical and histopathological outcome in our xenograft mouse model and included ^18^F-FDG PET studies in the first part of our mouse experiments.

## Materials and methods

### Cells and reagents

The A-204 human MRT cell line (which is falsely claimed to be a rhabdomyosarcoma cell line) was obtained from the American Type Culture Collection (ATCC; Rockville, MD) and maintained in a complete culture medium (McCoy) supplemented with 10% FCS. SAHA was obtained from Alexis Biochemicals (Lörrach, Germany), DMSO from Carl Roth Biochemicals (Karlsruhe, Germany). Ketamine (0.4 mg/20 g BW) and xylazine (Bayer Germany) (90 mg/20 g BW) were used to anesthetize mice during radiation treatments.

### Animal model

Xenografts of human MRT cells were established by subcutaneous inoculation of 5 × 10^6 ^A-204 cells into the hind legs of 10 weeks old female BALB/cNu/Nu athymic mice (Charles River, Wilmington, Mass.). The mice were maintained under specific pathogen-free conditions, food and water were supplied ad libitum. Housing and all procedures involving the mice were performed according to the protocols approved by the local regional board.

Two trial cohorts were treated with slight differences in the treatment regimen.

In our first cohort mice were randomly assigned to the different treatment groups, when tumors reached 300 mm^3 ^as PET-imaging prior to therapy was only possible in rather big tumors. In our second xenograft cohort we set aside PET-imaging and assigned the tumors to the different treatment arms when they reached 100 mm^3^.

Cohort I consisted of 12 mice per treatment group, Cohort II of 10 mice per treatment group.

The four treatment arms were as follows:

1) Vehicle (DMSO) control,

2) SAHA alone (100 mg/kg for 8 days (first cohort of mice) or 21 days (second cohort of mice)

3) XRT alone (with 1 × 10 Gy in the first cohort and 3 × 3 Gy on 3 consecutive days in the second cohort) and Combination treatment of SAHA with XRT (correspondingly, Cohort 1:

4) SAHA for 8 days + 1 × 10 Gy XRT, Cohort II: SAHA for 15 days (5 days/week for 3 weeks) + 3 × 3 Gy XRT).

Initially each group of the first cohort consisted of 13 mice. 1 mouse in the XRT group and 2 mice in the group with combination treatment died during anesthesia-related complications before XRT. In the second cohort each treatment group consisted of 10 mice.

All mice were sedated with ketamine/xylazine at the days, when PET-imaging or XRT were performed (day -1, 0 and day 8).

SAHA 100 mg/kg was solubilized in DMSO (99.5%), given intraperitoneally (i.p.), starting 24 h before XRT. The decision to start the SAHA treatment 24 h before XRT was based on our in vitro data that showed the highest sensitization of tumor cells after incubation for 24 hours [8]. On day 1, mice tumors were irradiated with a single dose of 10 Gy (Cohort I) and 3 × 3Gy (Cohort II), respectively, directed at the tumor site. This dose corresponds to a tumor growth delay of 20 days which was determined in a preceding test. During the follow-up mice were weighed, and the tumor sizes were measured using a calliper twice a week. Tumor length (L) and width (W) were measured and tumor volume calculated as (L × W^2^/2), where L = longest diameter and W = shortest diameter.

Animals were euthanized by CO_2 _inhalation followed by cervical dislocation when tumors reached 3.5 cm^3 ^or latest 65 days after treatment start.

### F-18 Fluorodeoxyglucose Positron Emission Tomography (^18^F-FDG-PET)

Dynamic ^18^F-FDG-PET measurements were performed in 5 mice per treatment group prior to treatment (day 0 for the untreated control group and XRT group, day -1 for the SAHA or SAHA plus XRT treated group) and at the end of treatment (day 8). The PET studies were performed in list mode for 60 min after intraveneous (i.v.) administration of 6 MBq ^18^F-FDG (in-house production, German Cancer Research Centre Heidelberg, Germany) using a matrix of 256 × 256 (pixel size 0.3882 × 0.3882 × 0.796 mm). There after images were reconstructed at definite time periods after tracer administration (2 × 15 s, 8 × 30 s, 5 × 60 s, 4 × 120 s, 2 × 210 s, 7 × 300 s). The images were reconstructed iteratively using the space alternating generalized expectation maximization method (SAGE, 16 subsets, 4 iterations) applying median root prior correction. Time-activity curves were created using volumes of interest (VOIs) over the hearts (input function) and the tumors. For the input function and the tissue response curve we used the maximal value of the VOI data. The following parameters were retrieved from the dynamic PET studies: standardized uptake value (SUV) at 50-60 minutes after tracer administration and the kinetic parameters k1, k2, k3, k4 and VB obtained form a pharmacokinetic analysis and the FDG influx calculated using the formula: influx = (k1*k3)/(k2 + k3). A dedicated micro PET system (Inveon, Siemens, Erlangen, Germany) was used for the animal studies. The evaluation of the PET-data was performed using the software package PMOD.

### Immunohistochemical analysis

Histomorphological analysis was done in three tumor samples of each treatment group of Cohort I on day 8 (corresponding to the last day of SAHA treatment) and at the last day of observation (day 60 at the latest). In Cohort II histological samples were taken on day 21 (corresponding to the last day of SAHA application in this Cohort). Tumors excised from euthanized mice were formalin-fixed and paraffin-embedded. 1 μM thick sections were cut and stained with hematoxylin-eosin (HE; Sigma-Aldrich, St Louis, USA). Subsequently, 3 μM thick sections were cut for immunohistochemistry. Staining for Ki-67 and Myo-D1 was performed as follows: First, tissue slides were deparaffinated with xylol and ethanol. To improve antigen retrival slides were cooked in 0.1 M citrate. Primary antibodies (monoclonal mouse anti-MyoD1 clone 5.8 A (1:50, over night, 4°C; Dako, Hamburg, Germany), monoclonal mouse anti-human Ki-67 clone MIB-1 (1:200, overnight 4°C; Dako, Hamburg, Germany)) were added. Then, a biotinylated anti-mouse secondary antibody was administered for 25 min. Endogenous peroxidase was blocked with H_2_O_2 _for 7 min. Slides were incubated with a streptavidin HRP construct (25 min,, Biolegend, San Diego, USA). Subsequently, slides were stained with 3-Amino-9-ethylcarbazole (AEC; Sigma-Aldrich, St Louis, USA) and counterstained with hemalaun (AppliChem, Darmstadt, Germany).

Apoptosis was quantified by the in situ apoptosis detection kit, ApopTAG^® ^(S7100; Chemicon International (Millipore), Temecula, CA, USA) according to the manufacturer's instructions.

All tissue evaluation was done by two trained pathologists (WW and AS).

H&E stainings were evaluated with respect to tumor cell pleomorphism, number of mitotic cells and extent of necrosis, estimated visually by an experienced pathologist. Ki-67 and Myo-D1 staining were quantified by Spectrum™ software (Aperio, Vista CA, USA).

TUNEL-positive cells were counted in ten high power fields of each case and a mean was calculated.

### Flow cytometry

γH2AX expression was assessed in three xenografts per treatment group using flow cytometry. Tumor cells were separated in a solution of 2.1% citrate acid and 0.5% tween. Tumor cells of three mice per group were washed with PBS several times and then fixed with 3% paraformaldehyde (PFA, Sigma) for 10 min at 37°C. Ice-cold methanol (90%) was added and samples were kept on ice for another 30 min. Afterwards, samples were washed three times in 0.5% PBS/BSA, resuspended in 100 μl 0.5% PBS/BSA and incubated for 10 min at RT. To stain the cells for γH2AX, the antibody (anti-phospho-Histone H2AX (Ser 139), clone JBW301) from Millipore (Molsheim, Germany) was diluted 1:10 in 0.5% PBS/BSA and 100 μl solution was applied per sample. After 1 h incubation time at RT, cells were washed another three times in PBS/BSA. Cells were further stained with DAPI for 30 min on ice. The samples were analyzed directly on a "BD™LSRII "- flow cytometer from "Becton, Dickinson and Company (Franklin Lakes, New Jersey US)". The relative fluorescence intensity in the gated areas was detected using the multiparameter "BD FACSDiva™" from "Becton, Dickinson and Company (Franklin Lakes, New Jersey US)" and analysed with the software "FlowJo7.6.5" by "Tree Star Inc". To assess the mean extent of DNA damage at a particular phase of the cell cycle, the mean values of γH2AX immunofluorescence (IF) were calculated separately for G_0/1_, S and G_2_M cells by the computer-interactive "gating" analysis. Cells in S and G_2_M have a 1.5 respectively 2.0 higher γH2AX mean IF compared to cells in G_0/1 _because of the increase of DNA and histone content during the cell cycle. Therefore, the data has to be normalized for DNA (histone) content by dividing the mean γH2AX IF of S- and G_2_M-phase cells by 1.5 and 2.0, respectively. Finally, a low level of γH2AX IF is seen in the untreated cells which represent an "intrinsic" γH2AX phosphorylation. Therefore, the γH2AX IF level of the untreated controls has to be subtracted from the IF level of the treated cells in order to get the γH2AX IF level which is treatment-related.

### Data analysis

Actual tumor growth delay was calculated with (T'_x_-T_x_)/T_x _as the time taken for the irradiatied tumors (T') and the control tumors (T) to x-fold multiply their volume (x).

The two-sided *t*-test was used to analyze the differences between the treatment groups. All data are presented as the mean +/- standard deviation. *P *values < 0.05 were considered statistically significant. Local Control (LC) was defined as the time from the initiation of treatment until the time a tumor had reached ≥ 1000 mm^3 ^in size. LC was estimated by Kaplan-Meier curves, and the differences in TTF between groups were assessed using log-rank test.

## Results

It was previously shown that SAHA is able to enhance radiosensitivity in MRT cells in vitro [8,9]. To extend these findings, we now investigated the in vivo radiosensitizing potential of SAHA in a MRT xenograft mouse model.

During the first 5 days of SAHA treatment, the animals suffered from loss of appetite. Even though the mice recovered soon application of SAHA was therefore not continued consecutively, but discontinued during weekends in the second cohort and well tolerated. Mice were weighed twice a week, body weight remained tolerable (maximum weight loss was 8% and similar in all treatment groups.

**Figure 1:**
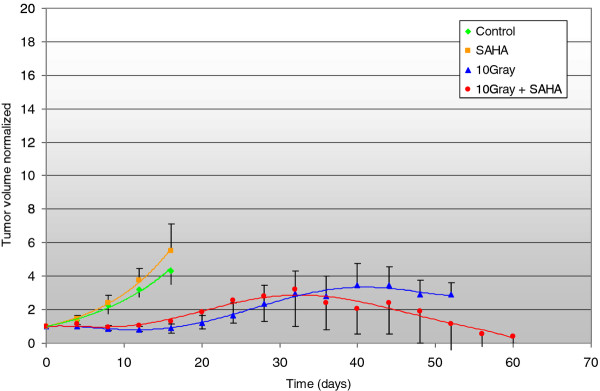
**Tumor progression of malignant rhabdoid tumor xenografts (MRT) in Cohort I after treatment with ionizing radiation (XRT) or SAHA and XRT**. The y-axis plots the multiplication of initial tumor size normalized to the initial size = 1, x-axis plots the time after treatment initiation in days. Figure 1 represents Cohort I and Figure 2 Cohort II. SAHA 100 mg/kg was injected intraperitoneally once daily for 8 consecutive days in Cohort I and for 15 days within 3 weeks in Cohort II starting with day 0. XRT with a single dose of 10 Gy (Cohort I) or with 3 × 3 Gy on three consecutive days (Cohort II) was delivered day 1(-3). After a follow-up of > 40 days, there was a trend towards slower progression in the combined treatment group compared to xenotransplants treated with XRT alone in Cohort I (*p *= 0.3), and a significant difference in Cohort II.(*p *< 0.05).

**Figure 2:**
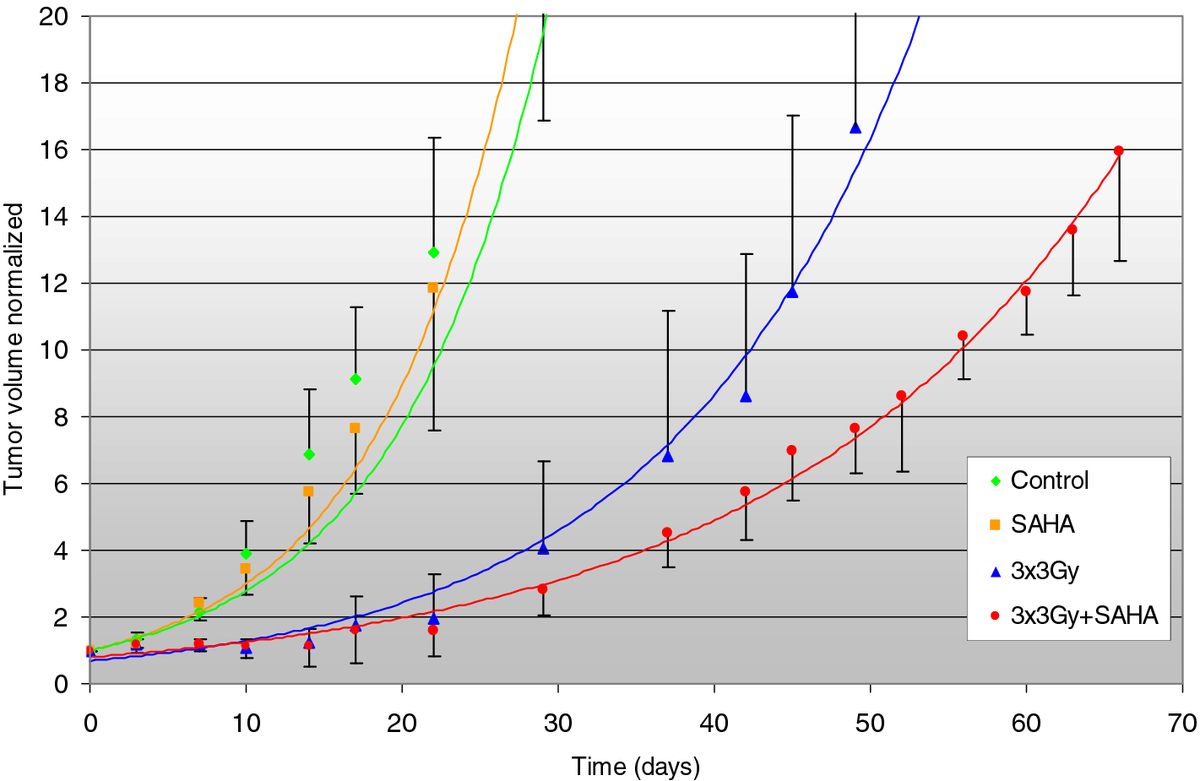
**Tumor progression of malignant rhabdoid tumor xenografts (MRT) in Cohort II after treatment with ionizing radiation (XRT) or SAHA and XRT**. The y-axis plots the multiplication of initial tumor size normalized to the initial size = 1, x-axis plots the time after treatment initiation in days. Figure 1 represents Cohort I and Figure 2 Cohort II. SAHA 100 mg/kg was injected intraperitoneally once daily for 8 consecutive days in Cohort I and for 15 days within 3 weeks in Cohort II starting with day 0. XRT with a single dose of 10 Gy (Cohort I) or with 3 × 3 Gy on three consecutive days (Cohort II) was delivered day 1(-3). After a follow-up of > 40 days, there was a trend towards slower progression in the combined treatment group compared to xenotransplants treated with XRT alone in Cohort I (*p *= 0.3), and a significant difference in Cohort II.(*p *< 0.05).

**Figure 3:**
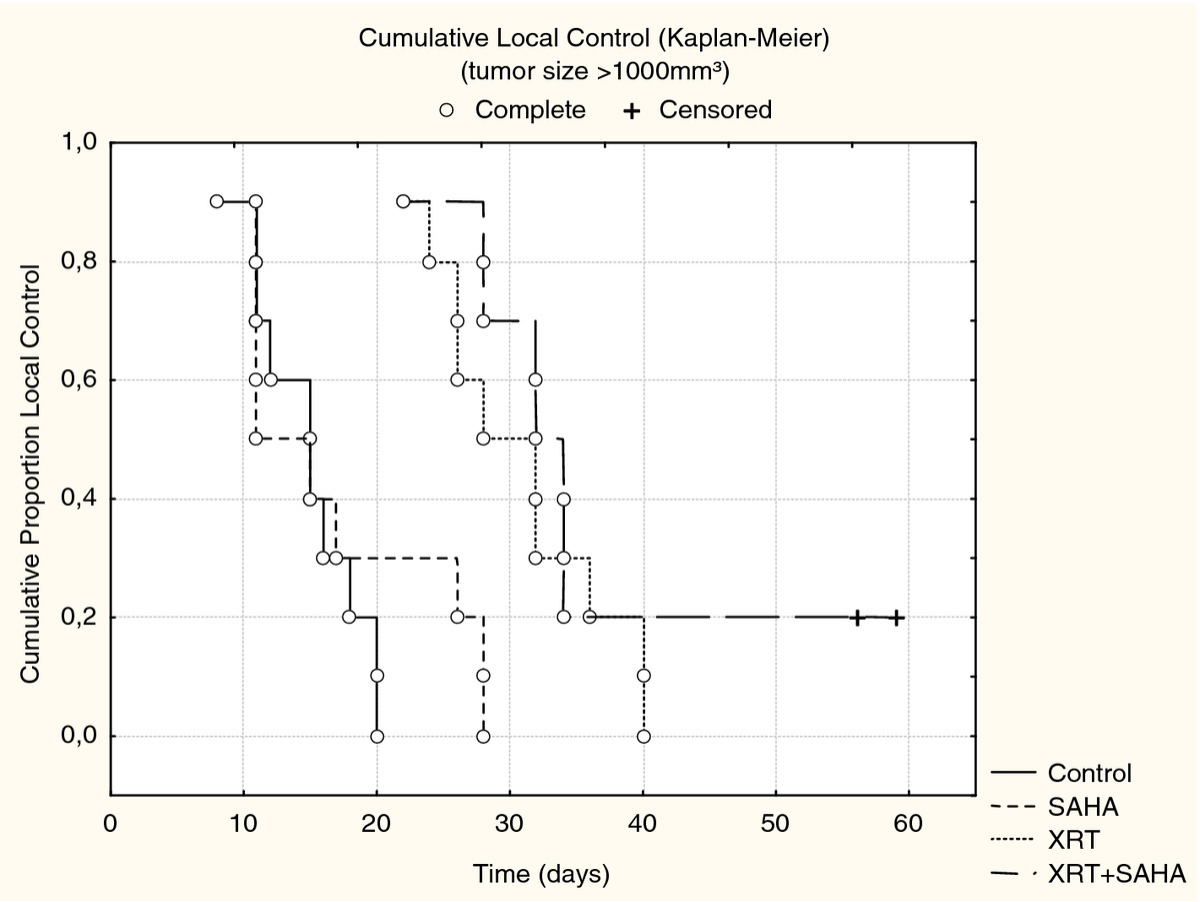
***Presents In vivo* tumor growth of rhabdomyosarcoma (RMS) xenografts after treatment with 10 Gy single dose radiation (XRT) or XRT and SAHA for 8 days (Cohort I)**. Local failure was defined as a tumor growth > 1000 mm^3^. Local Control was calculated according to the method of Kaplan and Meier. Fifty days after treatment start.

**Figure 4:**
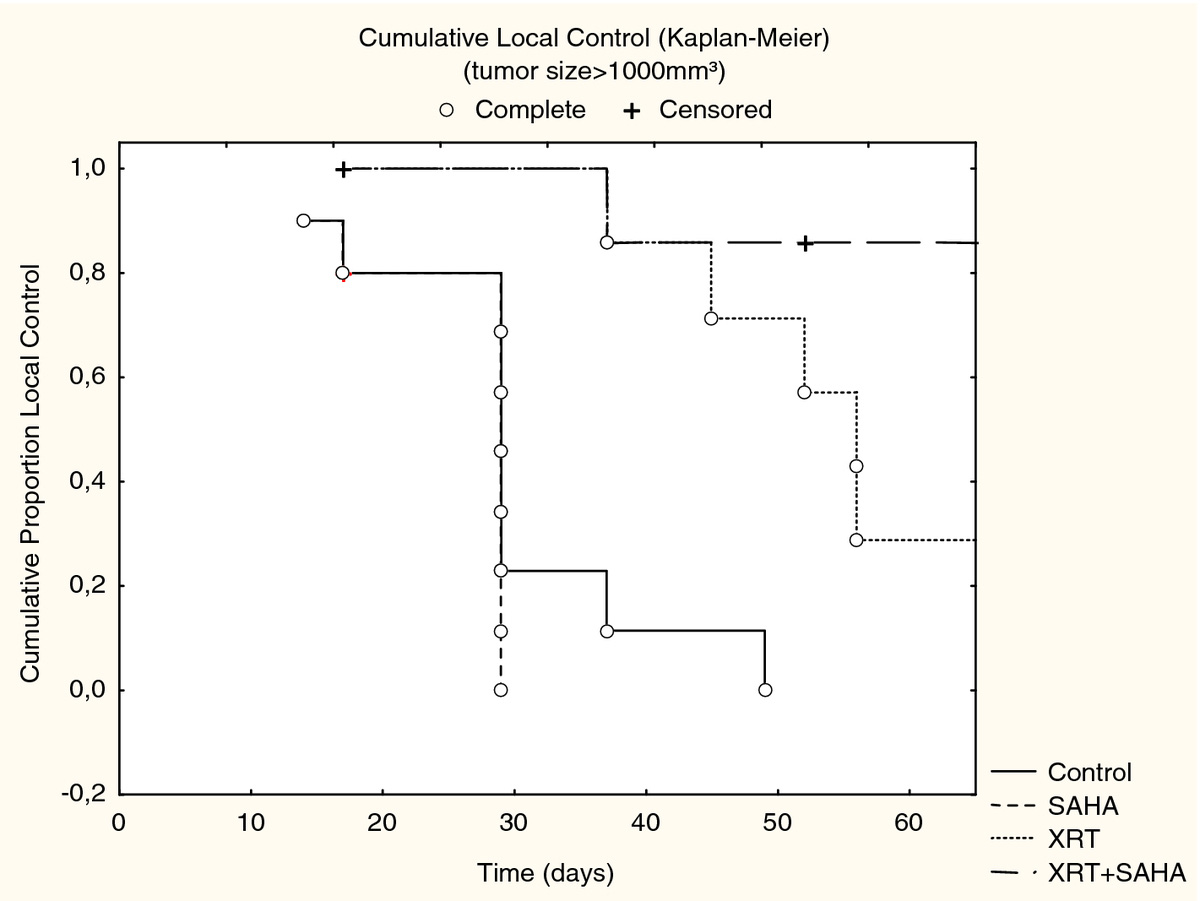
***Presents In vivo *tumor growth of rhabdomyosarcoma (RMS) xenografts after treatment with 3 × 3Gy fractionated radiation or XRT and SAHA for 3 weeks (Cohort II)**. Local failure was defined as a tumor growth > 1000 mm^3^. Local Control was calculated according to the method of Kaplan and Meier. Fifty days after treatment start.

Treatment with XRT respectively XRT plus SAHA delayed the tumor growth rate compared to treatment with SAHA alone or untreated controls. After a follow-up of > 40 days our data showed a trend towards slower tumor progression in the combined treatment group compared to xenotransplants treated with XRT alone, but this was not statistically significant in Cohort I treated with single-dose XRT and SAHA for 8 days (*p *= 0.3) (Figure 1a). However, we were able to prove statistical significance in the second cohort, treated with 3 × 3Gy fractionated XRT and SAHA for 3 weeks (*p *< 0.05) (Figure 2) We calculated local control (absences of local failure defined as tumor growth > 1000 mm^3^) according to the method of Kaplan and Meier in both cohorts. In Cohort I 50 days after start of treatment 20% of mice in the combination group compared to 0% of mice in the XRT had not experienced local failure (Figure 3). In cohort II, in which treatment started when tumors were much smaller, the same trend was intensified with a local control rate of 85% versus 25% after 60 days in favor of the combined treatment group. SAHA as a single-agent had no influence on local control in our experimental setting, neither after being given for 8 days (Cohort I) nor for 3 weeks (Cohort II) (Figure 4).

**Figure 5:**
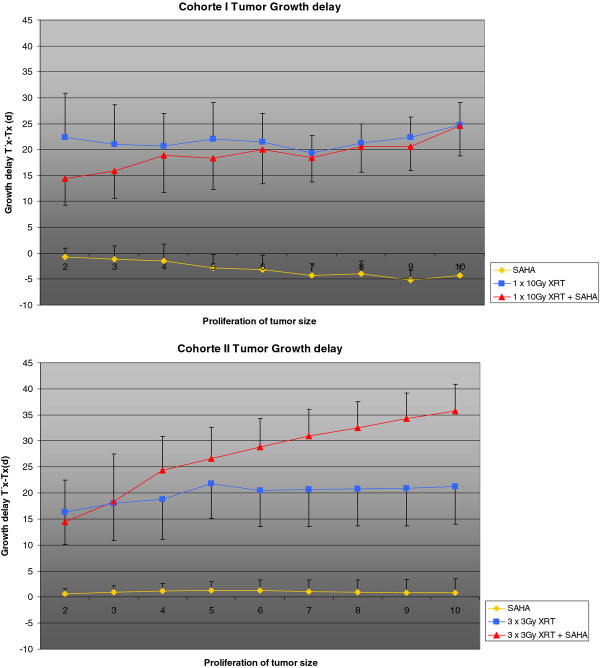
**Actual tumor growth delay was calculated with (T'_x_-T_x_)/T_x _as the time taken for the irradiatied tumors (T') and the control tumors (T) to x-fold multiply their volume (x)**. The y-Axis shows tumor growth delay in days, the x-axis shows the tumor growth (T'_x_-T_x_)/T_x_. Tumor growth delay was significant in Cohort II (*p *< 0.05), but not in our initial Cohort I.

Actual tumor growth delay was calculated with (T'_x_-T_x_)/T_x _as the time taken for the irradiatied tumors (T') and the control tumors (T) to x-fold multiply their volume (x). Tumor growth delay was significant in Cohort II (*p *< 0.05), in which the parameters initial tumor size, fractionation of radio-treatment and period of SAHA application had been changed, but not in our initial Cohort I (Figure 5).

**Figure 6:**
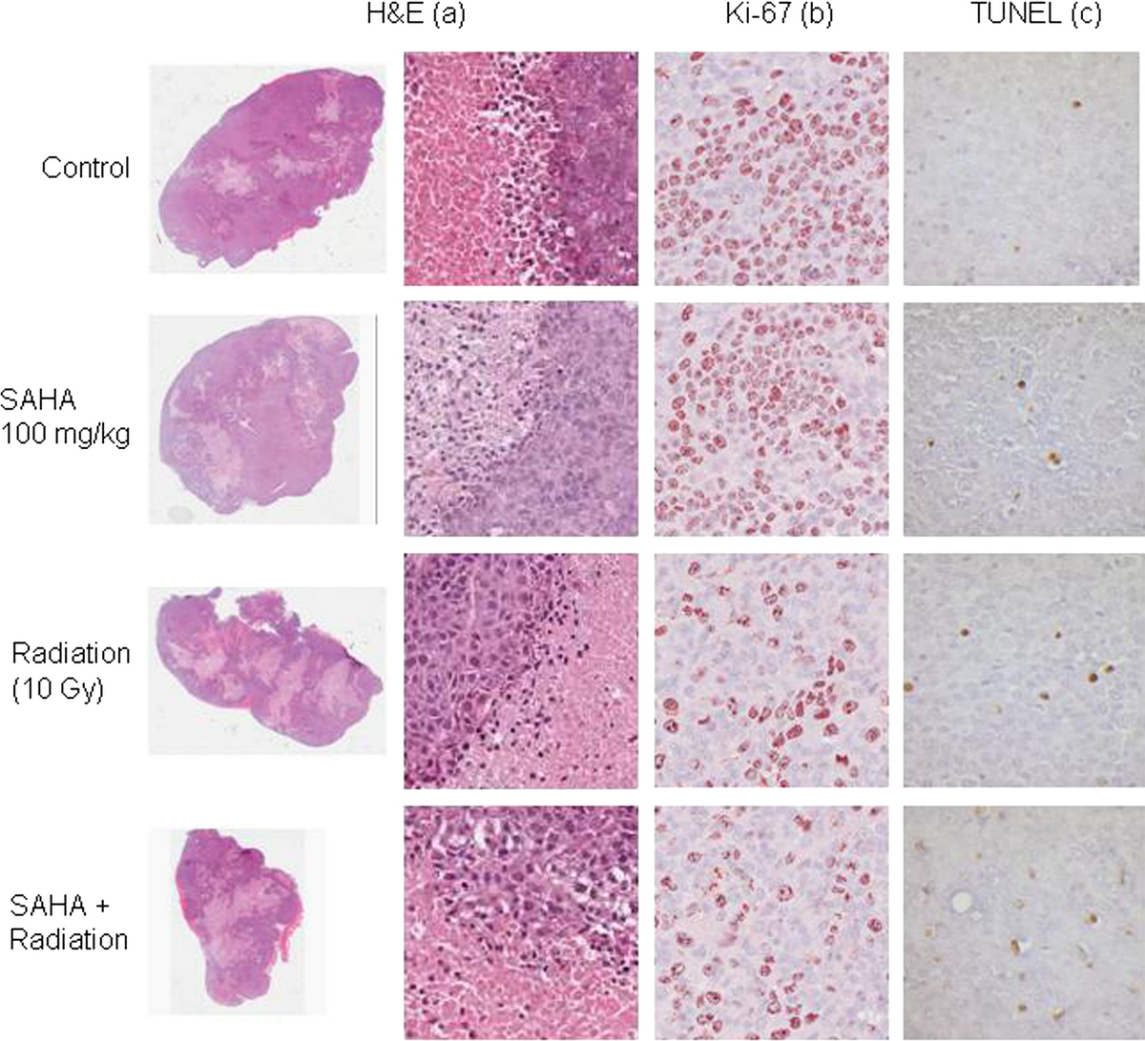
**H&E, TUNEL and Ki67 staining of tumor tissue from MRT xenografts treated with vehicle, suberoylanilide hydroxamic acid (SAHA), ionizing radiation (XRT) or SAHA and XRT**. See also Table 1.

At the last day of treatment (day 8 and day 21 respectively), necrosis was observed in 25% of control tumors in Cohort I and 8% of tumors in Cohort II. This difference was attributed to the difference in initial tumor size at the time treatment was started. Interestingly in both cohorts any treatment had no statistically significant impact on the induction of necrosis. (Table 1, Figure 6). The analysis of Ki-67 expression on day 8 as well as day 21 showed significantly higher proliferation rates (*p *< 0.03, *p *< 0.02) in the control tumors and the groups treated with SAHA alone compared to the groups treated with XRT ± SAHA. However, no statistically significant difference between the XRT and XRT + SAHA group was observed in either cohort (Table 1, Figure 6).

**Table 1:** Ki67-expression/Necrosis (%) and Apoptosis (TUNEL test) in RMS xenografts treated with vehicle (DMSO), suberoylanilide hydroxamic acid (SAHA), irradiation (XRT), or SAHA and XRT in the first and second mouse cohort observed day 8 (cohort I) and day 21 (Cohort II)

Treatment	Mean Tumor Age (days)		Mean (SD) Ki-67 (%)		HE (necrosis)(%)		TUNEL (apoptosis)	
	*Cohort I Cohort II*		*Cohort I CohortII*		*Cohort I CohortII*		*Cohort I CohortII*	
Control	39 (± 3)	37	42 (± 16)	45 (± 12)	25 (± 11)	8 (± 6)	64 (± 9)	55 (± 9)
SAHA	36 (± 1)	37	53 (± 10)	32 (± 11)	23 (± 9)	10 (± 6)	118 (± 15)	97 (± 12)
XRT	36 (± 1)	37	21 (± 4)	24 (± 7)	42 (± 12)	10 (± 5)	148 (± 24)	126 (± 12)
SAHA + XRT	36 (± 1)	37	14 (± 8)	17 (± 4)	48 (± 17)	12 (± 6)	201 (± 11)	192 (± 10)

Further, we investigated apoptosis on day 8 (cohort I) and day 21 (cohort II) in three tumors per treatment group by TUNEL test. Our results showed a significant increase of TUNEL positive cells in the tumors with combination treatment compared to tumors treated with XRT only (*p *= 0.03(cohort I) and *p *< 0.0016 (cohort II)) (Table 1).

**Figure 7:**
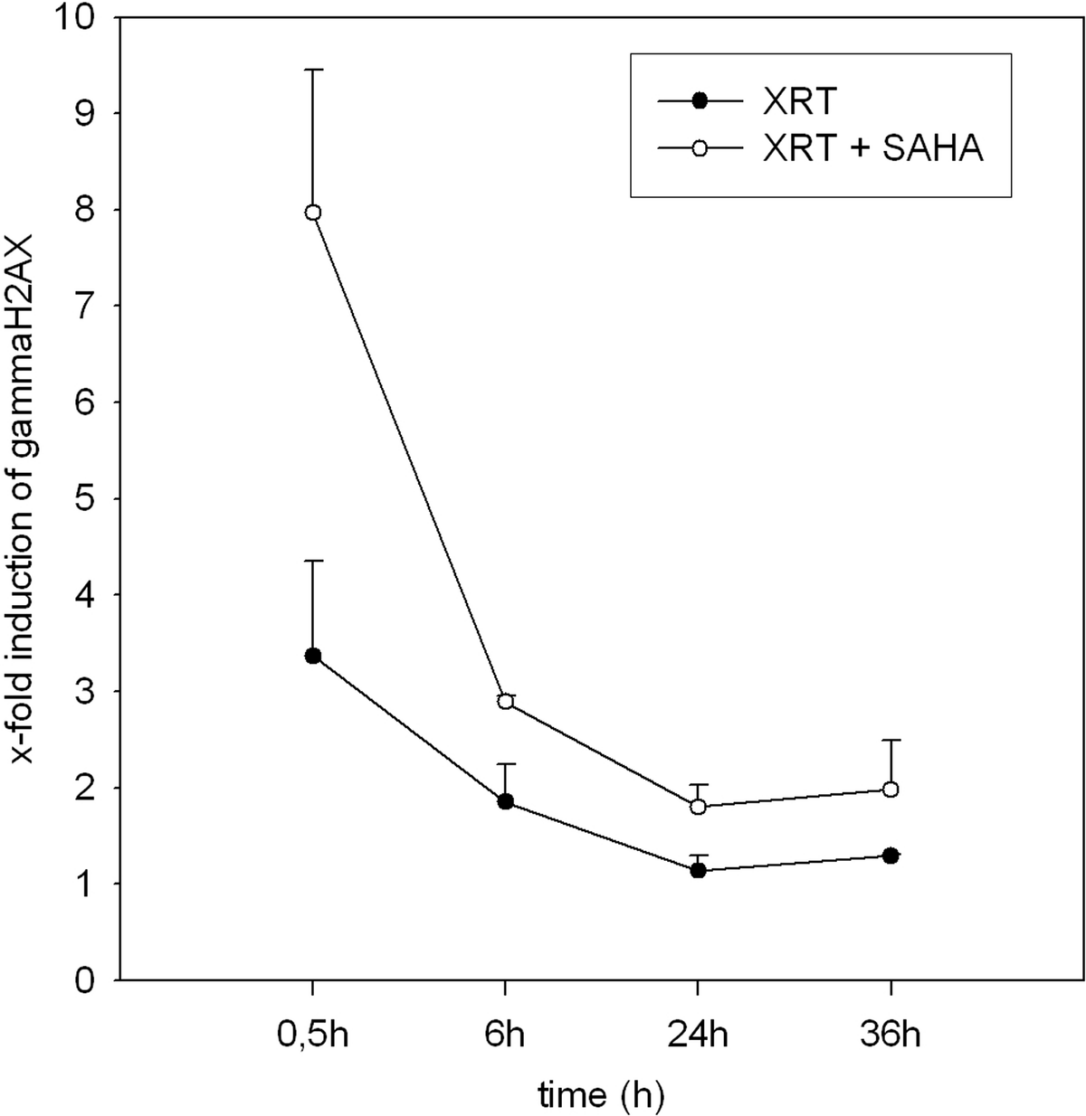
**γH2AX expression in MRT xenografts treated with ionizing radiation (XRT) or suberoylanilide hydroxamic acid (SAHA) and XRT**. The mice were treated with either vehicle control, SAHA 100 mg/kg for two consecutive days, XRT (10 Gy) or SAHA (100 mg/kg) on two consecutive days with XRT (10 Gy) on the second day of SAHA treatment. After 30 min, tumors of mice treated with XRT and SAHA showed a significantly higher induction of γH2AX expression compared to tumors treated with XRT alone. γH2AX expression decreased successively from 2 h to 6 h and 24 h after XRT. 24 h after XRT, γH2AX expression of irradiated tumors reached the level of the untreated controls whereas the tumors of the combination group showed still an elevated expression level.

We further evaluated induction of DSBs in the different treatment groups. It was previously demonstrated that DSB result in phosphorylation of H2AX on the γ-site of serine 139 to form γH2AX [12-14]. We quantified the γH2AX expression in the xenograft tumors of three tumors per treatment group. The mice were treated with either vehicle control, SAHA 100 mg/kg for two consecutive days, XRT (10 Gy) or SAHA (100 mg/kg) on two consecutive days with XRT (10 Gy) on the second day of SAHA treatment. The mice were euthanized 30 min, 6 h, 24 h and 36 h after treatment, tumors were excised and analyzed for γH2AX expression using flow cytometry. After 30 min, a single XRT dosage of 10 Gy resulted in a 3.4 (± 1)-fold induction of γH2AX expression compared to the vehicle control. Tumors of mice treated with XRT and SAHA showed a significantly higher induction (*p *= 0.03) of γH2AX expression (8 ± 1.5 -fold) compared to the vehicle control. SAHA given as a single agent resulted in no increase of γH2AX expression compared to the vehicle control (Figure 7). Investigation of further time points revealed that γH2AX expression decreased successively from 2 h to 6 h and 24 h after XRT. Interestingly 24 h after XRT, γH2AX expression of irradiated tumors reached the level of the untreated controls whereas the tumors of the combination group still showed an elevated expression level (2-fold ± 0.5).

Dynamic PET was used for tumor imaging to evaluate therapy response as previously described in animals after transfer of antiangiogenic genes [15]. In a preceding test four PET-tracers were tested in 6 mice with our sarcoma xenograft with every tracer being tested in 3 mice: FDG (^18^F-fluordeoxyglucose), FLT (^18^F-3'-deoxy-3'-L-thymidine), ^68 ^Ga-RGD (ανβ3/ανβ5 integrin-binding peptide) and FET (^18^F-fluoro-ethyl-tyrosine). ^18^F-FDG-PET showed the best results. Therefore, dynamic ^18^F-FDG-PET was performed in 5 mice per treatment group before and on day 8 after treatment with SAHA, XRT or XRT plus SAHA (Table 2).

**Table 2:** Descriptive statistics of the mean value and its standard deviation as well as the median values prior and after treatment of rhabdoid tumor xenografts with vehicle (DMSO), suberoylanilide hydroxamic acid (SAHA), irradiation (XRT) or XRT and SAHA

Parameter	Control		SAHA		XRT		SAHA + XRT	
	Mean (SD) prior	Mean (SD) after	Mean (SD) prior	Mean (SD) after	Mean (SD) prior	Mean (SD) after	Mean (SD) prior	Mean (SD) after
k1	0.015 (0.04)	0.13 (0.06)	0.16 (0.04)	0.1 (0.01)	0.14 (0.01)	0.16 (0.02)	0.14 (0.01)	0.15 (0.05)
k2	0.32 (0.07)	0.38 (0.02)	0.37 (0.12)	0.24 (0.08)	0.33 (0.03)	0.34 (0.02)	0.39 (0.03)	0.39 (0.02)
k3	0.08 (0.03)	0.11 (0.06)	0.07 (0.04)	0.1 (0.08)	0.06 (0.02)	0.08 (0.02)	0.08 (0.01)	0.09 (0.00)
VB	0.01 (0.001)	0.02 (0.002)	0.03 (0.002)	0.01 (0.001)	0.04 (0.001)	0.01 (0.001)	0.04 (0.003)	0.01 (0.001)
Influx constant Ki (=(k1xk3)/(k2+k3)	0.03	0.03	0.02	0.03	0.02	0.03	0.02	0.03
Max mSUV	1323 (368)	1593 (631)	2341 (1135)	729 (305)	1676 (782)	1578 (1206)	1314 (767)	1158 (816)

Comparing the results prior to and after treatment in each group there was no statistically significant effect on the standard uptake value (SUV) or on the transport of glucose into the tumor (k1) or out of the tumor (k2) in the different treatment groups. There was also no statistically significant impact on the hexokinase activity (k3) by any treatment modality examined. The vascular fraction (VB) increased in the control group, but equally decreased in all treated tumor groups. Therefore, FDG-PET did not seem to be an interesting option to monitor MRT treatment response in our mouse trial and was therefore discontinued after treatment of Cohort I.

## Discussion

This is the first in vivo study that shows, that vorinostat has the potential to sensitize MRT to radiation.

Patients with malignant rhabdoid tumors still have a poor prognosis despite intensive current treatment protocols. Radiotherapy plays a significant role in local treatment, but is often delayed as long as possible in young children as late sequelae are feared. Therfore there is a need for new treatment strategies.

Epigenetic targeting therapies with HDACi have shown promising results in different tumor types. SAHA is one of the well-established HDACi which has been approved in the United States by the Food and Drug Administration (FDA) for the therapy for human cutaneous T-cell lymphoma. The drug can be given orally and side-effects are minor compared to cytotoxic chemotherapeutics. Therefore, it is an interesting candidate for tumor therapy. The promise of HDACi for cancer treatment, as a single agent and in combination with standard therapies, is supported by in vitro results of colorectal carcinoma, human melanoma and glioma cell experiments [4,5,7]. In several trials combination of HDACi with chemotherapy or radiotherapy improved tumor cell kill [4,7,16,17]. However, in vivo studies with HDACi as single-agent in cancer treatment, showed only moderate and limited efficacy [18].

Based on our promising in vitro results that showed a selectively radiosensitizing effect in the A-204 cell line used in this experiment as well as in two osteosarcoma cell lines, we now investigated the efficacy of the HDACi SAHA in combination with radiotherapy, compared to SAHA or XRT only in a MRT xenograft mouse model.

The results of our first Cohort did show a trend towards an improved local control and tumor growth delay in mice treated with SAHA plus XRT compared to animals treated with XRT alone. We attributed the lack of significance in Cohort I to three possible parameters: 1. short period of SAHA application, 2. fractionation of radiation and 3. rather big tumor size at time of treatment initiation.

SAHA has previously been shown to augment the effects of radiotherapy in vivo tumor models [3,6,16]. In some of these in vivo studies, the drug was applied for several weeks with successful radiosensitization [7,19], even though in others short-term application for 5 days and even single-dose treatment prior to irradiation seemed to be sufficient [7,19].

The underlying pathways of HDACi acting as a radiosensitizer are still not completely understood [20]. Abrogation of DSB repair has been suggested to be one possible mechanism by several authors [10,16]. In our experiments, γH2AX expression, representing DNA-damage, was significantly influenced by the combination treatment. Interestingly, γH2AX expression of irradiated tumors reached the level of the untreated controls 24 h after XRT, whereas the tumors of the combination group showed still an elevated expression level (2-fold ± 0.5), thus showing an impairment of repair kinetics by the combination treatment. Therefore, we assumed improved results after fractionated XRT and decided to treat Cohort II with 3 × 3Gy instead of 1 × 10 Gy.

We wanted to use our first Cohort to investigate the potential of PET-imaging in our malignant rhabdoid tumor model. As the results were disappointing, we refrained from PET-imaging and thus were able to start treatment in smaller tumors in Cohort II.

These changes did indeed lead to a higher clinical impact resulting in significancy in Cohort II. As we decided to change all three parameters we can only guess which change has the highest impact and it remains unclear, if fractionation is obligatory or not. In previous reports on xenograft studies concerning breast tumors, neuroblastoma and ovarian cancer combination of HDACIs with fractionated [7,17] as well as single-dose irradiation [19] have been reported to be successful.

Interestingly - in contrast to reports concerning in vivo models of other tumor types [16,17,20]- application of SAHA as a single agent had no effect in both of our experimental designs in this MRT xenotransplant model - neither when given for 8 days, nor for 3 weeks. Our SAHA dose, especially in Cohort II with 100 mg/kg for 5 consecutive days for 3 weeks is rather high compared to other studies [7,17,19,20], in which SAHA proved to be successful. In these studies application differed from once 50 mg/kg, 3 × 150 mg within 1 week, 5 × 12,5 mg/week for 3 weeks and indeed 24 × 50 mg within 8 weeks. We therefore conclude from our study, that SAHA only treatment in MRT is not too promising.

We observed a strong trend towards a lower proliferation activity in the combination groups compared to the XRT alone groups. However, this trend failed to reach statistical significance in both cohorts.

Looking at necrosis of tumors, results showed no significant differences between the control group, SAHA group and the XRT or XRT plus SAHA group. However, apoptosis was indeed significantly higher in the XRT (*p *= 0,001) as well as XRT + SAHA groups (*p *= 0,0001) compared to the controls in both cohorts and also significantly higher in both XRT + SAHA groups compared to SAHA only (*p *> 0,0016). Thus, induction of apoptosis rather than necrosis seems to have the higher impact on radiosensitization by SAHA. However, as far as apoptosis was concerned the effect of SAHA + XRT seemed to be rather additive, while tumor growth delay in the mice seemed to be a rather synergistic effect given that SAHA alone had no significant effect at all. This proves again that apoptosis remains to be just one of the -all in all not completely understood - underlying mechanisms of radiosensitization.

We attribute the fact that no changes in our dynamic 18F-FDG-PET/CT images were to be observed to this lack of all investigated treatment schedules to induce significant necrosis in MRT. However, a further reason may be the initial tumor size (mean tumor size 300 mm3), which we chose in Cohort I in order to at all allow PET imaging [21], but which also may have attributed to the lower impact of the overall treatment that was observed in Cohort I compared to Cohort II.

## Conclusions

SAHA is a promising radiosensitizer in malignant rhabdoid tumors, while it has no significant effect as a mono-agent. The addition of SAHA to radiotherapy does favorably and significantly influence apoptosis and DNA-repair kinetics in MRT xenotransplants, whereas necrosis is not influenced.

## Competing interests

The authors declare that they have no competing interests.

## Authors' contributions

MT designed methods, carried out the experiments and analysed the data. SO co-designed the research theme, interpreted the results and wrote paper. VE helped to design of the flow-cytometry experiments and to interpret the results. WW and AS carried out the histopathological assessment in the tumor specimen and helped to interpret the results. MB and K-JW helped by discussing analyses, interpretation and presentation. RLP carried out and analyzed flow-cytometry assessment of y-H2AX experiments. UH designed and helped to analyze PET-experiments. AK co-designed the research theme. JD co-designed the research theme and provided the laboratory and radiation facilities. PEH co-analysed and co-discussed the data. CB designed the research theme, analysed and co-discussed all results. All authors contributed to and approved this manuscript.

## References

[B1] Buscariollo DL Park HS Roberts KB Survival outcomes in atypical teratoid rhabdoid tumor for patients undergoing radiotherapy in a Surveillance Epidemiology, and End Results analysis Cancer 2011 in press 10.1002/cncr.27373 22213196

[B2] Morgenstern DA Gibson S Brown T Clinical and pathological features of paediatric malignant rhabdoid tumours Pediatr Blood Cancer 2010 51 1 29 34 19653294 10.1002/pbc.22231

[B3] Graham JS Kaye SB Brown R The promises and pitfalls of epigenetic therapies in solid tumors Eur J Cancer 2009 45 1129 1136 10.1016/j.ejca.2009.01.003 19211243

[B4] Folkvord S Ree AH Furre T Radiosensitization by SAHA in experimental colorectal carcinoma models - *in viv *effects and relevance of histone acetylation status Int J Radiat Oncol Biol Phys 2009 74 2 546 552 10.1016/j.ijrobp.2009.01.068 19427556

[B5] Munshi A Kurland JF Nishikawa T Histone deacetylase inhibitors radiosensitize human melanoma by suppressing DNA repair activity Clin Cancer Res 2005 11 4912 4922 10.1158/1078-0432.CCR-04-2088 16000590

[B6] Chinnaiyan P Vallabhaneni G Armstrong E Modulation of radiation response by histone deacetylase inhibition Int J Radiation Oncology Biol Phy 2005 62 223 229 10.1016/j.ijrobp.2004.12.088 15850925

[B7] Entin-Meer M Yang X Vandenburg SR *In viv *efficacy of a novel histone deacetylase inhibitor in combination with radiation for the treatment of gliomas Neuro Oncol 2007 9 82 88 10.1215/15228517-2006-032 17347490 PMC1871664

[B8] Blattmann C Oertel S Ehemann V Enhancement of radiation response in osteosarcoma and rhabdomyosarcoma cell lines by histone deacetylase inhibition Int J Radiat Oncol Biol Phys 2010 78 237 245 10.1016/j.ijrobp.2010.03.010 20646843

[B9] Knipstein JA Birks DK Donson AM Histone deacetylase inhibition decreases proliferation and potentiates the effect of ionizing radiation in atypical teratoid/rhabdoid tumor cells Neuro Oncol 2012 in press 10.1093/neuonc/nor208 PMC3266389 22156471

[B10] Munshi A Tanaka T Hobbs ML Vorinostat, a histone deacetylase inhibitor, enhances the response of human tumor cells to ionizing radiation through prolongation of gamma-H2AX foci Molecular Cancer Therapy 2006 5 1967 1974 10.1158/1535-7163.MCT-06-0022 16928817

[B11] Denecke T Hundsdörfer P Misch D Assessment of histological response of paediatric bone sarcomas using FDG PET in comparison to morphological volume measurement and standardized MRI parameters Eur J Nucl Med Mol Imaging 2010 37 1842 1853 10.1007/s00259-010-1484-3 20505933

[B12] Paull TT Rogakou EP Yamazaki V A critical role for histone H2AX in recruitment of repair factors to nuclear foci after DNA damage Curr Biol 2000 10 886 895 10.1016/S0960-9822(00)00610-2 10959836

[B13] Huang X Darzynkiewicz Z Cytometric assessment of histone H2AX phosphorylation: *Method* Mol Biol 2006 314 73 80 10.1385/1-59259-973-7:073 PMC1458374 16673875

[B14] Olive PL Banath JP Phosphorylation of histone H2AX as a measure of radiosensitivity Int J Radiation Oncology Biol Phys 2003 58 331 335 10.1016/j.ijrobp.2003.09.028 14751500

[B15] Haberkorn U Hoffend J Schmidt K Changes in glucose metabolism and gene expression after transfer of anti-angiogenic genes in rat hepatoma Eur J Nucl Med Mol Imaging 2007 34 12 2011 2023 10.1007/s00259-007-0520-4 17701172

[B16] Lopez G Liu J Ren W Combining PCI-24781, a novel histone deacetylase inhibitor, with chemotherapy for the treatment of soft tissue sarcoma Clin Cancer Res 2009 15 19 3472 3483 19417021 10.1158/1078-0432.CCR-08-2714

[B17] Mueller S Yang X Sottero TL Cooperation of the HDAC inhibitor vorinostat and radiation in metastatic neuroblastoma: efficacy and underlying mechanisms Cancer Lett 2011 306 2 223 229 10.1016/j.canlet.2011.03.010 21497989 PMC3244077

[B18] Siu LL Pili R Duran I Phase I study of MGCD0103 given as a three-times-per-week oral dose in patients with advanced solid tumors J Clin Oncol 2008 26 1940 1947 10.1200/JCO.2007.14.5730 18421048 PMC3501257

[B19] Baschnagel A Russo A Burgan WE Vorinostat enhances the radiosensitivity of a breast cancer brain metastatic cell line grown in vitro and as intracranial xenografts Mol Cancer Ther 2009 8 6 1589 1595 10.1158/1535-7163.MCT-09-0038 19509253 PMC3393105

[B20] Chen MY Liao WS Lu Z Decitabine and suberoylanilide hydroxamic acid (SAHA) inhibit growth of ovarian cancer cell lines and xenografts while inducing expression of imprinted tumor suppressor genes, apoptosis, G2/M arrest, and autophagy Cancer 2011 16 DOI: 10.1002/cncr.26073 10.1002/cncr.26073 PMC3137708 21491416

[B21] Arvanitis C Bendapudi PK Tseng JR (18)F and (18)FDG PET imaging of osteosarcoma to non-invasively monitor in situ changes in cellular proliferation and bone differentiation upon MYC inactivation Cancer Biol Ther 2008 7 12 1947 1951 10.4161/cbt.7.12.6947 18981708 PMC4158945

